# Supplementary material for: Parallel evolution of amphioxus and vertebrate small-scale gene duplications
Source: Genome Biol. 2022 Nov 18;23:243. doi: 10.1186/s13059-022-02808-6 (PMC9673378; doi:10.1186/s13059-022-02808-6)
Supplement: Supplementary file 4 — Additional file 4. Review history. [file 13059_2022_2808_MOESM4_ESM.docx]

**Review history**

**First round of review**

**Reviewer 1**

This manuscript by Brasó-Vives et al. reports a greatly improved assembly of the genome of the European lancelet (Branchiostoma lanceolatum) and presents an comparative analysis of independent gene duplication between the 3 genera of Branchiostoma with assembled genomes and vertebrates. The results reveal similar levels of independent gene duplications in vertebrates and lancelets and lower rates of gene duplication in "house-keeping" genes such as mitochondrial and transcriptional functions than for genes involved in the immune system or cell-signaling. The results are convincing and the manuscript well-written. However, the figures are not at all clear for any reader not steeped in bioinformics. For Figure 1, I had to find a GO browser to find out what "molecular function" and "biological function" genes are. The definition of "molecular function" (A molecular process that can be carried out by the action of a single macromolecular machine, usually via direct physical interactions with other molecular entities. Function in this sense denotes an action, or activity, that a gene product (or a complex) performs. These actions are described from two distinct but related perspectives: (1) biochemical activity, and (2) role as a component in a larger system/process) is meaningless. Only the list of the 29, 277 "molecular function" genes (e.g. antioxidant activity, ATP-dependent activitybindingcargo receptor activity, etc.) makes sense. I found a list of genes with biological functions in Supplementary Table 4. That table might also have a list of "molecular function" genes, but after scrolling through the first ~1000 genes, I tired of infinite scrolling. Such a complete table is useful, but most readers will want to gain a reasonable idea of the genes in each category in the text itself. In other words, the authors should give the reader enough information in the text itself so that the reader knows more or less what the authors are talking about. The many, many supplemental figures and tables are good for the person deeply interested in the details, but they are quite overwhelming. Most readers will want to grasp the take-home message from the text alone. The first Figure 4 on p. 13 is quite confusing as it claims to show duplicated gene expression in B. lanceolatum relative to vertebrates. But then it has bars for both single copy genes (solid) and for duplicated genes (hatched) but on the X-axis both solid and hatched bars correspond to single copy genes or to ohnologs etc. I think perhaps the authors are comparing single copy and duplicated genes in the lancelet to single copy genes in vertebrates, and then to ohnologs in vertebrates etc. However, gene expression is apparently determined from RNA-Seq. Did the authors dissect tissues from the lancelet and compare gene expression in individual tissues to their corresponding tissues in vertebrates? Which tissues and which embryonic stages are being compared. For example, how can gene expression in a lancelet notochord be compared to that in adult vertebrate notochords (i.e. intervertebral discs)? Then, there is the problem that in the pdf version of the text, Figure 5 is labeled Figure 4.
In sum, this is a valuable study, but the authors should have someone who is not thoroughly steeped in bioinformatics or lancelet/amphioxus research read the manuscript without the supplemental data and ensure that he/she can understand both the text and especially the figures. If they can't explain a difficult subject to the general reader interested in comparative genomics, they will lose much of the intended audience.

**Reviewer 2**

The manuscript reports a new version of the B. lanceolatum genome assembly and annotation and, together with 6 other known chordate genomes, exploits this new data to examine the evolution of duplicate genes. The main finding is that small-scale duplications tend to have occurred in the same genes in the amphioxus lineage as they have in the vertebrate lineage.

I like how the investigation does not rely on phylogenetic tree reconstructions but rather on orthogroups. Indeed, while reconciled gene trees do position duplication nodes, these tend to be notoriously hard to place accurately and often result in flawed interpretations. Here the authors took a more generic and safe approach, perhaps at the cost of less resolution but this is not an issue for their investigation.

If the conclusions hold, I think that the results are clear and would provide a neat reference for the notion that duplicated genes are retained due to common forces in independent lineages.

I have one only one major issue with the conclusions, and I fear that it is quite fundamental, so I'd be really happy if it could be disproved. As the authors well know, when a similar character or trait (here duplication patterns) is found in two divergent lineages, the first scenario that should come to mind is that this character is ancestral to the two lineages, i.e. it happened once and got inherited. This scenario uses parsimony as a guiding principle, which is not always correct but generally considered the null hypothesis because it involves fewer events than any other scenario.

Here, the analysis finds that many genes that duplicated in the amphioxus lineage also duplicated in the vertebrate lineage. What if a simple reason for it is that they in fact duplicated in the stem chordate lineage? The authors may have many good reasons to think that this was not the case, but if so, I am surprised that this parsimonious scenario did not get one single mention in the manuscript. Without a clear rebuttal of this parsimonious scenario, I believe that we cannot accept the author's conclusions that the duplication patterns are there because of independent duplications under similar kinds of constraints.

The fact that the analysis does not include external groups to chordates complicates the interpretation, leaving the two scenarii (parsimony vs independence) theoretically equally likely (of note, depending of what is found in an elusive outgroup, it could only disprove independence, not prove shared-by-descent). Of course, I understand that external groups are not easy to find for chordates, leaving few options.

One way to test the alternatives is perhaps to look in early diverging vertebrates (hagfish, lamprey, perhaps Chondrichthyes) and see if the pattern is absent, which would add weight to the author's scenario.

Also, the authors rapidly dismiss the tunicate's lineage from their analysis because of its fast evolutionary rate, but even the more so, if they see the same pattern as in amphioxus and vertebrate in this rapidly diverging lineage, this may strengthen their conclusions (there are > 5200 orthogroups with both a human and a Ciona gene in Ensembl, providing plenty enough data to test this). However, at a stretch, one might argue that a similar pattern in tunicates may also derive from the same chordate ancestral state as in amphioxus and vertebrates. In any case it would add to a very interesting discussion.

The argument that lineage-specific duplications behave one way or another cannot be used to argue for or against the author's scenario (I think) because these could equally result either from lineage specific losses in the other branch, or indeed lineage specific duplications.

A second important issue I think is that we absolutely need to see a dot-plot matrix with the position of amphioxus paralogs in the 3 species (similar to Suppl. Fig 6, but only within one species vs itself in each plot). This is a basic sanity check that would reveal immediately if transposable elements, repeatedly amplified pseudogenes or other such non-coding genes have been incorrectly included in the set. They would show a biased distribution pattern, and if absent, would eliminate these sources of artefact from the analysis.

I have a stream of more minor points I'm afraid, because many parts of the manuscript, especially pertaining to Figure 2 that illustrates the core result of the manuscript, need extensive clarifications.

1. L 85 : I think perhaps the introduction, which is nicely written but stays at a very general level, should better outline what to expect with regards to shared/diverging patterns between amphioxus and vertebrates. Is it that surprising that the patterns are similar after all? Also it would be useful to the reader if the introduction was more specific about what known duplication rates are in the relevant lineages, how they compare, and therefore how dissimilar we might expect the vertebrate and amphioxus lineages to be. Also don't vertebrate display a hugely diverse rate of duplication rates and functional patterns themselves, making it hard to generalize about a "vertebrate rate and pattern"?


2. L96: Please specify "27,102 protein-coding genes"

3. L128: please define PHD

4. L144: "This is, gene duplications … " awkward sentence construction?

5. Table 2 reports 25,946 genes in B. lanceolatum but the authors mention 27,102 annotated genes Line 96. This number does not correspond to the subset of 26,468 genes with "strong evidence" (L465) either, although methods Line 485 indicates that this is the set that was used for orthogroups.

6. The text mentions orthogroups "with duplications", i.e. orthogroups with more than one gene, of one or more species, inside. But Table 2, and at other places in the text (eg L182-183, L206), there is mention of "Duplicated orthogroups" which is confusing because it suggests a duplication at the root of the orthogroup, which is not what the authors mean (I think). It would be good to change "Duplicated" to "with duplications" in the relevant header of the Orthogroup class in Table and in the text.


7. L187: "two types of duplication mechanisms". Which two types? The text only mentions one just above (tandem duplications). This refers to my point about the introduction, which would be improved if more focus would be placed on duplications, their mechanism and their functional implications.

8. L198: "Gene duplication patterns are conserved between amphioxus and vertebrates". But isn't the point of Figure 2D to show that duplication patterns between B. lanceolatum and vertebrates are significantly different? I interpret Figure 2D as follows: there is a significant excess of single-copy B. lanceolatum genes compared to vertebrate single copy genes (bottom left of Fig. 2D), there is a significant deficit of B. lanceolatum single copy genes among vertebrate small-scale duplicates (bottom right of Fig. 2D) and there is a significant excess of B. lanceolatum small-scale duplicates among vertebrate small-scale duplicates. What am I getting wrong? I guess the authors should clarify what is the "expected" rate when calculating the Fold Change for Figure 2D.

9. L220: I do not understand "both vertebrate small-scale duplicates and ohnologs contribute to the general correlation of constraints on duplication in functional categories…". The text just proposed that the functional correlation could also be due to a "tolerance to duplication", not a constraint.

10. Figure 3: I am not sure I understand how to read Figure 3. In Fig. 3A, a point is a % of single copy genes in human and in B. lanceolatum. So a point at coordinates 50-50 for example, means that 50% of single copy human genes (about half of human genes according to Table 2) belong to a GO category that also encompasses 50% of B. lanceolatum single-copy genes? Is this correct? If so, does it make sense to include such generic GO terms in the analysis?

11. Figure 3: why are the % of amphioxus genes systematically higher than their human counterparts in all Figure 3 panels. In other words, all points are above the x=y diagonal. This is difficult to understand, given that B. lanceolatum genes are either single-copy or duplicated. Does this mean that there is a significantly higher proportion of GO-labelled genes in B. lanceolatum in general and a deficit of GO-labelled genes in human)? This is unexpected given that the GO-terms originally come from the human gene set.

12. Figure 4B and 4D: embryonic, not embrionic

13. L295: "B. lanceolatum and B. floridae diverged between 20 and 150 million years ago (Mya) but share around 500 My of common evolution since their lineage split with that of other chordates" is confusing. The uncertainty in the split between the two amphioxus lineage should also apply to the stem branch going back to the chordate ancestor, since the split defines both the branch to the present and the branch to the past.

14. L300. Chromosomal splits are called fissions


15. L319: "Overall, although inter-chromosomal duplications have been frequent in the amphioxus lineage, small-scale duplications are found with more probability within the same chromosome." This is not at all apparent from Supp Figure 6. On the contrary, for a given chromosome, the sum of homologs in the other species seems much larger across all the other chromosomes than on the homologous chromosome, even if the latter does concentrate duplicates because of the one-to-many and tandem duplicate categories.

16. L322: the authors do not comment on the striking similarity between B. lanceolatum chromosomes 10 and 12 against chicken chromosomes. It seems typical of a situation where either the two chromosomes in amphioxus are the result of a relatively recent fission, or the chromosomes ancestral to 10 and 12 fused in the chicken lineage before being somewhat scattered in the extant chicken genome. Given the remarkable stability of the chicken genome, the former scenario is much more likely.

Methods:

17. L486: I do not understand how there could be mismatches between cDNA (in fact, coding sequences, CDS) and protein sequences since protein sequences are derived from CDS, themselves derived from the coding exon sequence annotated in genomic DNA. On another note, I really think that the term "cDNA" should be reserved for actual complementary DNA sequences resulting from reverse transcription of a mRNA.

18. L499: please clarify what amphioxus/vertebrate means. Amphioxus _or_ vertebrate but not both?

**Reviewer 3**

Overall I find that this is an interesting paper that makes a valuable contribution to the field. The figures could be made clearer and more reader-friendly, and there are a couple of aspects of the manuscript as it currently stands that I don't think add much and which I think either need substantial additional work to be more robust, or could be removed without negatively impacting the contribution of the current paper.

There are several points that I think need reconsideration or alternative presentation.

Major points:

I think there should be a more cautious approach in the interpretation of the apparently 'lineage-specific' amphioxus genes. There are apparently 4559 of these compared to 8705 orthogroups. The authors correctly list three possible explanations for these apparently lineage-specific genes - gene loss in vertebrates; de novo origin; homology detection failure (HDF). De novo origin is probably a small fraction of these considering the large numbers, so instead these probably mostly represent HDF and gene loss in the genomes studied. The latter could be investigated by inclusion of more genomes, though the authors might decide that is too much additional work. Importantly, HDF would normally be found in fast-evolving genes, and these may have other biases or trends in terms of evolutionary properties and functional categories. For these reasons, I would recommend extreme caution in the interpretation of these apparent lineage specific genes. I would suggest that the text should be modified to emphasise this and also that they should be removed from some parts of the analysis because I would have little faith that any robust conclusions can be drawn from them without conducting additional tests to separate out gene loses from de novo from HDF. This applies to most/all mentions of the lineage specific genes throughout the text.

p8 line 181 - it is stated that zebrafish has a higher proportion of orthogroups with duplications, possibly due to the teleost WGD, but shouldn't this also result in a higher mean # of genes in the duplicated orthogroups, yet table 2 shows that mouse has a higher mean than zebrafish? This needs to be better explained.

p10 - considering the overlap in duplications between amphioxus and vertebrates does it not naturally follow that there is also overlap in functional categories, especially when GO terms are inferred through orthology? I'm not sure what I can learn from this section as it is not sufficiently clear.

It takes some effort to understand the figures and I think that more could and should be done to make them a little more reader-friendly. The legends could be more informative, and the figure design could perhaps be made clearer too. For example Figure 3 is very difficult to interpret and even after spending some time to figure it out, I wasn't sure how I should interpret it. What does this show? How robust is it? I am not sure that it adds anything and perhaps this whole section could be dropped or radically revised.

p12 line 255 - "On the other had, the lower expression and higher specificity ..." this sentence is a bit strange to me. I am not sure what it is intended to convey. My first instinct was that these characteristics might simply reflect the process of dup gene preservation (ie subfunc) which indeed is argued later on the same page. However, this sentence might mislead the reader to think that the authors mean that lower expression/tissue specific genes are more likely to be duplicated in the first place, which is not what the data shows - I think this second half of the sentence should be deleted (from "proposes secondary but more specialised roles ..." onwards) because that is the part that I find puzzling ... or it should be rephrased.

Fig 4 is hard to read - especially part E - this needs better description in the legend and a clearer graphical presentation.


Minor points

p3 line 71 - "Notably to understand the role of duplication in vertebrates we need to characterise their role in the reference outgroup lineage." -- In what sense? What new can we learn with this reference point considering the amount of data we already have within vertebrates? I think this current work has intrinsic value in terms of understanding amphioxus evolution, so it isn't necessary, to me, to justify this paper by stating it is useful for understanding vertebrate duplications. But if you do want to claim this then I think you should explain yourself.

p4 line 85: "Surprisingly" -- why is it a surprise that genome average rates of nucleotide substitution are not correlated with gene duplication? Has this been studied previously? What were the patterns?

p10 line 212: 'positive relation' is ambiguous - please rephrase

p14 line 329 - The sentence doesn't make sense. Is it supposed to be "more frequent THAN in the vertebrate lineage"?

**Authors’ response to reviewers**

Reviewer #1:
This manuscript by Brasó-Vives et al. reports a greatly improved assembly of the genome of the European lancelet (Branchiostoma lanceolatum) and presents an comparative analysis of independent gene duplication between the 3 genera of Branchiostoma with assembled genomes and vertebrates. The results reveal similar levels of independent gene duplications in vertebrates and lancelets and lower rates of gene duplication in "house-keeping" genes such as mitochondrial and transcriptional functions than for genes involved in the immune system or cell-signaling. The results are convincing and the manuscript well-written. However, the figures are not at all clear for any reader not steeped in bioinformatics. For Figure 1, I had to find a GO browser to find out what "molecular function" and "biological function" genes are. The definition of "molecular function" (A molecular process that can be carried out by the action of a single macromolecular machine, usually via direct physical interactions with other molecular entities. Function in this sense denotes an action, or activity, that a gene product (or a complex) performs. These actions are described from two distinct but related perspectives: (1) biochemical activity, and (2) role as a component in a larger system/process) is meaningless. Only the list of the 29, 277 "molecular function" genes (e.g. antioxidant activity, ATP-dependent activity binding cargo receptor activity, etc.) makes sense. I found a list of genes with biological functions in Supplementary Table 4. That table might also have a list of "molecular function" genes, but after scrolling through the first ~1000 genes, I tired of infinite scrolling. Such a complete table is useful, but most readers will want to gain a reasonable idea of the genes in each category in the text itself. In other words, the authors should give the reader enough information in the text itself so that the reader knows more or less what the authors are talking about. The many, many supplemental figures and tables are good for the person deeply interested in the details, but they are quite overwhelming. Most readers will want to grasp the take-home message from the text alone. The first Figure 4 on p. 13 is quite confusing as it claims to show duplicated gene expression in B. lanceolatum relative to vertebrates. But then it has bars for both single copy genes (solid) and for duplicated genes (hatched) but on the X-axis both solid and hatched bars correspond to single copy genes or to ohnologs etc. I think perhaps the authors are comparing single copy and duplicated genes in the lancelet to single copy genes in vertebrates, and then to ohnologs in vertebrates etc. However, gene expression is apparently determined from RNA-Seq. Did the authors dissect tissues from the lancelet and compare gene expression in individual tissues to their corresponding tissues in vertebrates? Which tissues and which embryonic stages are being compared. For example, how can gene expression in a lancelet notochord be compared to that in adult vertebrate notochords (i.e. intervertebral discs)? Then, there is the problem that in the pdf version of the text, Figure 5 is labeled Figure 4.
In sum, this is a valuable study, but the authors should have someone who is not thoroughly steeped in bioinformatics or lancelet/amphioxus research read the manuscript without the supplemental data and ensure that he/she can understand both the text and especially the figures. If they can't explain a difficult subject to the general reader interested in comparative genomics, they will lose much of the intended audience.

Response:
We appreciate and thank the reviewer for the valuable comments and the time dedicated to review our manuscript.
We understand that although the use of GO terms is very common in bioinformatic research, it might, indeed, not be straightforward and accessible for a reader not steeped in bioinformatics. We have clarified the definitions of GO “molecular function” and “biological processes” categories as well as extended the explanation about the results obtained in this part of the manuscript.
Regarding the supplementary figures and tables, they are meant to be of use of the readers interested in the details. We agree that the main text alone should be enough to adequately describe our results and convey our conclusions. We have, hence, made an effort to clarify the results in the main text and have made sure that supplementary data remains complementary and not essential to the manuscript. We have also redesigned most of the figures in the main text to make them more accessible, and easier to read and understand. We agree that they needed much improvement and we again thank the reviewer for the suggestions.
Regarding Figure 4, we have used published gene expression data from B. lanceolatum tissues and compared it to gene expression data in homologous tissues in zebrafish [1]. The selection of homologous tissues follows from the previous work done by some of our coauthors [1], which was done for ohnolog genes only. We here have extended it to both types of duplicated genes (adding small-scale duplicates in both lineages). We have clarified the figure and its explanation in the main text. We thank the reviewer for pointing out our previous lack of clarity.
The typographical error on the labeling of Figure 5 has been fixed (it is now Figure 6).
Reviewer #2:
The manuscript reports a new version of the B. lanceolatum genome assembly and annotation and, together with 6 other known chordate genomes, exploits this new data to examine the evolution of duplicate genes. The main finding is that small-scale duplications tend to have occurred in the same genes in the amphioxus lineage as they have in the vertebrate lineage.

I like how the investigation does not rely on phylogenetic tree reconstructions but rather on orthogroups. Indeed, while reconciled gene trees do position duplication nodes, these tend to be notoriously hard to place accurately and often result in flawed interpretations. Here the authors took a more generic and safe approach, perhaps at the cost of less resolution but this is not an issue for their investigation.

If the conclusions hold, I think that the results are clear and would provide a neat reference for the notion that duplicated genes are retained due to common forces in independent lineages.

I have one only one major issue with the conclusions, and I fear that it is quite fundamental, so I'd be really happy if it could be disproved. As the authors well know, when a similar character or trait (here duplication patterns) is found in two divergent lineages, the first scenario that should come to mind is that this character is ancestral to the two lineages, i.e. it happened once and got inherited. This scenario uses parsimony as a guiding principle, which is not always correct but generally considered the null hypothesis because it involves fewer events than any other scenario.

Here, the analysis finds that many genes that duplicated in the amphioxus lineage also duplicated in the vertebrate lineage. What if a simple reason for it is that they in fact duplicated in the stem chordate lineage? The authors may have many good reasons to think that this was not the case, but if so, I am surprised that this parsimonious scenario did not get one single mention in the manuscript. Without a clear rebuttal of this parsimonious scenario, I believe that we cannot accept the author's conclusions that the duplication patterns are there because of independent duplications under similar kinds of constraints.

The fact that the analysis does not include external groups to chordates complicates the interpretation, leaving the two scenario (parsimony vs independence) theoretically equally likely (of note, depending on what is found in an elusive outgroup, it could only disprove independence, not prove shared-by-descent). Of course, I understand that external groups are not easy to find for chordates, leaving few options.

One way to test the alternatives is perhaps to look in early diverging vertebrates (hagfish, lamprey, perhaps Chondrichthyes) and see if the pattern is absent, which would add weight to the author's scenario.

Response:
This is a very reasonable concern, and we agree that it should be addressed to confirm the manuscript results. So far, we didn’t consider this issue because we relied on the orthogroup calling algorithm, Broccoli [2], which is specially designed to split orthogroups with duplications more ancient than the last common ancestor of all the considered species.

Figure 1B from Derelle et al. 2020 [2] (above) corresponds to an example of Broccoli’s orthogroup’s building “species overlap” criteria used to evaluate the two sets of leaves emerging from each node in a gene tree and decide if they belong to the same orthogroup or not. In the example of the figure, each node is evaluated, and only the nodes in orange pass the criteria. For this reason the algorithm will group all genes within the orange rectangle (emerging from the oldest node passing the criteria) within the same orthogroup and exclude the other genes (that is, the three to the right of the figure will be grouped in a different orthogroup). This criterion is designed to classify genes resulting from duplications older than the last common ancestor of all considered species in different orthogroups and not in the same.
According to this orthogroup building criteria, duplications older than the last common ancestor between vertebrates and amphioxus are not considered in our analysis (or they are considered in different orthogroups) and do not impact our results. Nevertheless, since we understand the importance of disproving the older duplication hypothesis as it directly impacts the results of our analysis, we have now used an independent and well established method of gene tree reconstruction to evaluate our orthogroups.
We have used MAFFT to build multiple sequence alignment of all protein sequences of genes in each orthogroup and RAxML (model PROTGAMMAAUTO) to build gene trees for each orthogroup. From the total number of orthogroups that are small-scale duplicates both in vertebrates and in amphioxus, RAxML was able to reconstruct 69.14% of the orthogroup’s trees, from which 79.95% had all vertebrate and amphioxus genes in two different monophyletic groups. This means that using a completely independent approach, we were able to prove that a large proportion of the orthogroups considered independent small-scale duplications in the vertebrate and amphioxus branches are indeed independent duplications and not ancient duplications. Similar results were obtained when considering orthogroups which have ohnologs in vertebrates and small-scale duplicates in amphioxus (66.37% of calculated trees and 74.83% of monophyletic grouping of both vertebrate and amphioxus genes).
In summary, the orthogroup building algorithm splits ancient duplications in different orthogroups, avoiding the concerning scenario. Still, in order to reinsure our results, we have used an independent method validating 80% of the concerned cases (small-scale duplicates in both branches). We hope that with this new evidence, the reviewer’s major concern is solved and our results validated. We have added the additional analysis and results to the manuscript. We are thankful to the reviewer for pointing out this issue, which has led to more robust results.

Also, the authors rapidly dismiss the tunicate's lineage from their analysis because of its fast evolutionary rate, but even the more so, if they see the same pattern as in amphioxus and vertebrate in this rapidly diverging lineage, this may strengthen their conclusions (there are > 5200 orthogroups with both a human and a Ciona gene in Ensembl, providing plenty enough data to test this). However, at a stretch, one might argue that a similar pattern in tunicates may also derive from the same chordate ancestral state as in amphioxus and vertebrates. In any case it would add to a very interesting discussion.
Response:
For this specific work we wanted to focus on the genome and evolutionary history of amphioxus taking advantage of the high quality genome reference that we present. This is why we decided to restrict our analysis to the amphioxus lineage, adding only good quality vertebrate model species genomes to the amphioxus ones. We think that including Ciona, for which we still lack a high quality reference genome, and whose genome is known to be very derived, will add a lot of noise and technical challenges to the study. We consider that adding Ciona would represent a completely different study including a non-negligible amount of work.

The argument that lineage-specific duplications behave one way or another cannot be used to argue for or against the author's scenario (I think) because these could equally result either from lineage specific losses in the other branch, or indeed lineage specific duplications.

A second important issue I think is that we absolutely need to see a dot-plot matrix with the position of amphioxus paralogs in the 3 species (similar to Suppl. Fig 6, but only within one species vs itself in each plot). This is a basic sanity check that would reveal immediately if transposable elements, repeatedly amplified pseudogenes or other such non-coding genes have been incorrectly included in the set. They would show a biased distribution pattern, and if absent, would eliminate these sources of artefact from the analysis.
Response:
We thank the reviewer for suggesting this plot. It adds another layer of information that we were not processing in the previous version of our manuscript. We have added the B. lanceolatum paralog synteny plot to the Supplementary Figure S6. It supports the results already seen in the B. lanceolatum, B. floridae paralog synteny plot. That is, small-scale duplicated genes have happened frequently and both in the same chromosome (intrachromosomal) and in different chromosomes (interchromosomal). Local expansions of genes are frequently found among B. lanceolatum paralogs, but are rarer among paralogs between B. lanceolatum and B. floridae suggesting a dynamic paralog synteny.

I have a stream of more minor points I'm afraid, because many parts of the manuscript, especially pertaining to Figure 2 that illustrates the core result of the manuscript, need extensive clarifications.

1. L 85 : I think perhaps the introduction, which is nicely written but stays at a very general level, should better outline what to expect with regards to shared/diverging patterns between amphioxus and vertebrates. Is it that surprising that the patterns are similar after all? Also it would be useful to the reader if the introduction was more specific about what known duplication rates are in the relevant lineages, how they compare, and therefore how dissimilar we might expect the vertebrate and amphioxus lineages to be. Also don't vertebrate display a hugely diverse rate of duplication rates and functional patterns themselves, making it hard to generalize about a "vertebrate rate and pattern"?
Response:
We knew very little about the duplication history in the amphioxus lineage, except for the absence of whole-genome duplications (WGD). This is why it is difficult to specify expectations in this regard. What we do know is that vertebrate genomes have both ohnologs and small-scale duplicates while amphioxus genomes, in the absence of WGD, only have small-scale duplicates. Given this difference and the important contribution that 2R ohnologs have in the vertebrate gene pool, our null hypothesis would be to find substantial differences in the evolutionary histories of these two lineages. It was surprising to find out that they share a lot of similarities and that there are core evolutionary constraints on duplication that seem to be conserved. We have extended our introduction to clarify what it is known about the duplication patterns in both lineages and what it is expected from them. Thank you for noting the lack of clarity.

Regarding the diverse rate of duplication of vertebrates, the reviewer is right, they show a big diversity of duplication rates. In this study, we were interested in focussing on the general trends of duplication common to all vertebrates, including their 2R whole genome duplications. For this reason, we included four different vertebrate species with very different evolutionary histories. We have now made this objective clearer in our introduction.

2. L96: Please specify "27,102 protein-coding genes"
Response:
Done.

3. L128: please define PHD
Response:
Done.

4. L144: "This is, gene duplications … " awkward sentence construction?
Response:
Modified to clarify the whole section.

5. Table 2 reports 25,946 genes in B. lanceolatum but the authors mention 27,102 annotated genes Line 96. This number does not correspond to the subset of 26,468 genes with "strong evidence" (L465) either, although methods Line 485 indicates that this is the set that was used for orthogroups.
Response:
Thank you for pointing this out. We had not properly described an additional filtering that we had carried out for the orthology analysis. From the 26,468 genes, 522 genes were filtered out after making sure that the gene annotation and the protein sequence were matching, in order to avoid potential non-coding genes spuriously annotated as coding (numbers for all species are in Table S6). We have now clarified this filter in methods and results.

6. The text mentions orthogroups "with duplications", i.e. orthogroups with more than one gene, of one or more species, inside. But Table 2, and at other places in the text (eg L182-183, L206), there is mention of "Duplicated orthogroups" which is confusing because it suggests a duplication at the root of the orthogroup, which is not what the authors mean (I think). It would be good to change "Duplicated" to "with duplications" in the relevant header of the Orthogroup class in Table and in the text.
Response:
Done. Thank you for the suggestion.

7. L187: "two types of duplication mechanisms". Which two types? The text only mentions one just above (tandem duplications). This refers to my point about the introduction, which would be improved if more focus would be placed on duplications, their mechanism and their functional implications.
Response:
Clarified.

8. L198: "Gene duplication patterns are conserved between amphioxus and vertebrates". But isn't the point of Figure 2D to show that duplication patterns between B. lanceolatum and vertebrates are significantly different? I interpret Figure 2D as follows: there is a significant excess of single-copy B. lanceolatum genes compared to vertebrate single copy genes (bottom left of Fig. 2D), there is a significant deficit of B. lanceolatum single copy genes among vertebrate small-scale duplicates (bottom right of Fig. 2D) and there is a significant excess of B. lanceolatum small-scale duplicates among vertebrate small-scale duplicates. What am I getting wrong? I guess the authors should clarify what is the "expected" rate when calculating the Fold Change for Figure 2D.
Response:
Following this comment, we realized the extremely complicated interpretation of our previous Figures 2C and 2D. We have redesigned these figures (now in Figure 2B) in order to make explicit what is the expected distribution of vertebrate orthologs among B. lanceolatum orthogroups and the difference with the observed numbers. We have also modified the text (both main text and figure caption) in order to facilitate the interpretation of the figure. Thank you for pointing this out.

9. L220: I do not understand "both vertebrate small-scale duplicates and ohnologs contribute to the general correlation of constraints on duplication in functional categories…". The text just proposed that the functional correlation could also be due to a "tolerance to duplication", not a constraint.
Response:
Clarified. Thank you for pointing out the inconsistency.

10. Figure 3: I am not sure I understand how to read Figure 3. In Fig. 3A, a point is a % of single copy genes in human and in B. lanceolatum. So a point at coordinates 50-50 for example, means that 50% of single copy human genes (about half of human genes according to Table 2) belong to a GO category that also encompasses 50% of B. lanceolatum single-copy genes? Is this correct? If so, does it make sense to include such generic GO terms in the analysis?
Response:
We have adapted Figure 3 to make it easier to understand, as well as its figure caption.

11. Figure 3: why are the % of amphioxus genes systematically higher than their human counterparts in all Figure 3 panels. In other words, all points are above the x=y diagonal. This is difficult to understand, given that B. lanceolatum genes are either single-copy or duplicated. Does this mean that there is a significantly higher proportion of GO-labelled genes in B. lanceolatum in general and a deficit of GO-labelled genes in human)? This is unexpected given that the GO-terms originally come from the human gene set.
Response:
We thank the reviewer for pointing this out. It is an observation worth discussing. As there is no GO annotation for BraLan3, we extrapolated the human GO annotation to B. lanceolatum meaning that all amphioxus genes orthologs to a given human gene with a given GO annotation were assigned to this GO term. In order to avoid biases in our results due to this extrapolation, we applied the inverse reasoning assigning all human orthologs to a B. lanceolatum gene assigned to a given GO term to this GO term and restricted the analysis to the human-B. lanceolatum orthologs. We think that, after these corrections, the reason why we see Figure 3 points mainly below (above in the previous version of the figure) the diagonal is because of the different distribution of duplicate genes across B. lanceolatum and H. sapiens orthogroups. H. sapiens has ~3% more duplicate genes in its genome compared to amphioxus, but more importantly, has ~5% more duplicated orthogroups than amphioxus (see Table 2). This results in a mean number of genes in duplicated orthogroups being 1.5 times bigger in B. lanceolatum. Overall, we think that this slight tendency is a consequence of the combination of the true distribution of duplicated genes across both species genomes and our strategy of GO annotation extrapolation and that it has no impact on our results. Again, we are thankful to the reviewer for pointing it out.

12. Figure 4B and 4D: embryonic, not embrionic
Response:
Corrected.

13. L295: "B. lanceolatum and B. floridae diverged between 20 and 150 million years ago (Mya) but share around 500 My of common evolution since their lineage split with that of other chordates" is confusing. The uncertainty in the split between the two amphioxus lineage should also apply to the stem branch going back to the chordate ancestor, since the split defines both the branch to the present and the branch to the past.
Response:
Clarified in the text.

14. L300. Chromosomal splits are called fissions.
Response:
Corrected, thank you for pointing this out.

15. L319: "Overall, although inter-chromosomal duplications have been frequent in the amphioxus lineage, small-scale duplications are found with more probability within the same chromosome." This is not at all apparent from Supp Figure 6. On the contrary, for a given chromosome, the sum of homologs in the other species seems much larger across all the other chromosomes than on the homologous chromosome, even if the latter does concentrate duplicates because of the one-to-many and tandem duplicate categories.
Response:
True, simplified.

16. L322: the authors do not comment on the striking similarity between B. lanceolatum chromosomes 10 and 12 against chicken chromosomes. It seems typical of a situation where either the two chromosomes in amphioxus are the result of a relatively recent fission, or the chromosomes ancestral to 10 and 12 fused in the chicken lineage before being somewhat scattered in the extant chicken genome. Given the remarkable stability of the chicken genome, the former scenario is much more likely.
Response:
Thank you for noticing this similarity, it is actually also seen for B. lanceolatum chromosome 17 and can also be observed in 3 B. floridae chromosomes in Simakov et al. 2020 [3]. We have added a comment about it in the text.

Methods:

17. L486: I do not understand how there could be mismatches between cDNA (in fact, coding sequences, CDS) and protein sequences since protein sequences are derived from CDS, themselves derived from the coding exon sequence annotated in genomic DNA. On another note, I really think that the term "cDNA" should be reserved for actual complementary DNA sequences resulting from reverse transcription of a mRNA.
Response:
We agree that in theory, protein sequences have to match with translated annotated coding exonic sequence, but we find out it is not always the case due to mainly annotation errors. This is why we decided to filter out all non-matching cases for the orthology analysis. We have clarified the source of such non-matching cases in the text and avoided using the term cDNA since we agree it should be restricted to DNA complementary sequences to the mRNA.

18. L499: please clarify what amphioxus/vertebrate means. Amphioxus _or_ vertebrate but not both?
Response:
Clarified.


Reviewer #3:
Overall I find that this is an interesting paper that makes a valuable contribution to the field. The figures could be made clearer and more reader-friendly, and there are a couple of aspects of the manuscript as it currently stands that I don't think add much and which I think either need substantial additional work to be more robust, or could be removed without negatively impacting the contribution of the current paper.

There are several points that I think need reconsideration or alternative presentation.

Major points:

I think there should be a more cautious approach in the interpretation of the apparently 'lineage-specific' amphioxus genes. There are apparently 4559 of these compared to 8705 orthogroups. The authors correctly list three possible explanations for these apparently lineage-specific genes - gene loss in vertebrates; de novo origin; homology detection failure (HDF). De novo origin is probably a small fraction of these considering the large numbers, so instead these probably mostly represent HDF and gene loss in the genomes studied. The latter could be investigated by inclusion of more genomes, though the authors might decide that is too much additional work. Importantly, HDF would normally be found in fast-evolving genes, and these may have other biases or trends in terms of evolutionary properties and functional categories. For these reasons, I would recommend extreme caution in the interpretation of these apparent lineage specific genes. I would suggest that the text should be modified to emphasize this and also that they should be removed from some parts of the analysis because I would have little faith that any robust conclusions can be drawn from them without conducting additional tests to separate out gene loses from de novo from HDF. This applies to most/all mentions of the lineage specific genes throughout the text.
Response:
We understand the reviewer’s concerns about lineage-specific genes. It is true that we have not quantified or controlled for HDF and have centered our attention in the detectable orthologs. We have, in consequence, added a more detailed explanation of the origin of what our analysis classifies as lineage-specific genes. We have also modified all the text regarding lineage-specific genes, and have been careful with the conclusions extracted therein. We are thankful to the reviewer for pointing it out, we think that our manuscript is more accurate after these corrections.

p8 line 181 - it is stated that zebrafish has a higher proportion of orthogroups with duplications, possibly due to the teleost WGD, but shouldn't this also result in a higher mean # of genes in the duplicated orthogroups, yet table 2 shows that mouse has a higher mean than zebrafish? This needs to be better explained.
Response:
This is a good point, in order to avoid classifying 3R teleost ohnologs as small-scale duplications in vertebrates while focusing in 2R ohnologs, we considered as non-duplicated all orthogroups that, among vertebrates, were only duplicated in zebrafish and that were known to be retained in the 3R WGD (see methods for further details). That is, we retain as duplicated all zebrafish orthogroups that have been also duplicated in another vertebrate species (increasing the number of overall duplicated orthogroups in zebrafish with respect to the other vertebrate species) but do not consider as duplicated all 3R specific duplicated orthogroups (affecting the mean number of genes in duplicated orthogroups and making it more comparable to other vertebrate species). We have modified the text in order to avoid misinterpretations in the numbers of zebrafish duplicated genes and orthogroups. Thank you for pointing it out, it was necessary to make it more clear in the text in order to avoid misinterpretations.

p10 - considering the overlap in duplications between amphioxus and vertebrates does it not naturally follow that there is also overlap in functional categories, especially when GO terms are inferred through orthology? I'm not sure what I can learn from this section as it is not sufficiently clear.
Response:
Considering the parallelism in duplicated orthogroups between the two lineages, one could naturally hypothesize that there is also a parallelism in the amount of duplicated genes within functional categories. This is precisely what we demonstrate in Figure 3. The two results are not independent of each other, but they are two different points of view of the same biological trend that have slightly different consequences and interpretation. We think that both sections complement each other and make our results more robust and complete. We have clarified this in the text.

It takes some effort to understand the figures and I think that more could and should be done to make them a little more reader-friendly. The legends could be more informative, and the figure design could perhaps be made clearer too. For example Figure 3 is very difficult to interpret and even after spending some time to figure it out, I wasn't sure how I should interpret it. What does this show? How robust is it? I am not sure that it adds anything and perhaps this whole section could be dropped or radically revised.
Response:
We realized the difficulty in understanding our figures following this and similar comments from other reviewers. We have now done a complete redesign of the main figures and figure captions in order to make them more clear. We are very thankful to the reviewer for pointing this out. We hope that, after this big redesign, the figures are much clearer and the overall manuscript is more accessible.

p12 line 255 - "On the other hand, the lower expression and higher specificity ..." this sentence is a bit strange to me. I am not sure what it is intended to convey. My first instinct was that these characteristics might simply reflect the process of dup gene preservation (ie subfunc) which indeed is argued later on the same page. However, this sentence might mislead the reader to think that the authors mean that lower expression/tissue specific genes are more likely to be duplicated in the first place, which is not what the data shows - I think this second half of the sentence should be deleted (from "proposes secondary but more specialised roles ..." onwards) because that is the part that I find puzzling ... or it should be rephrased.
Response:
Clarified in the text.

Fig 4 is hard to read - especially part E - this needs better description in the legend and a clearer graphical presentation.
Response:
As stated above, we have done a complete redesign of all the figures, specially figure 4E and 4F (now figure 5).

Minor points

p3 line 71 - "Notably to understand the role of duplication in vertebrates we need to characterize their role in the reference outgroup lineage." -- In what sense? What new can we learn with this reference point considering the amount of data we already have within vertebrates? I think this current work has intrinsic value in terms of understanding amphioxus evolution, so it isn't necessary, to me, to justify this paper by stating it is useful for understanding vertebrate duplications. But if you do want to claim this then I think you should explain yourself.
Response:
Rephrased and clarified.

p4 line 85: "Surprisingly" -- why is it a surprise that genome average rates of nucleotide substitution are not correlated with gene duplication? Has this been studied previously? What were the patterns?
Response:
Better explained in the text.

p10 line 212: 'positive relation' is ambiguous - please rephrase
Response:
Rephrased.

p14 line 329 - The sentence doesn't make sense. Is it supposed to be "more frequent THAN in the vertebrate lineage"?
Response:
Rephrased.

1. Marlétaz F, Firbas PN, Maeso I, Tena JJ, Bogdanovic O, Perry M, et al. Amphioxus functional genomics and the origins of vertebrate gene regulation. Nature. 2018;564:64–70.
2. Derelle R, Philippe H, Colbourne JK. Broccoli: Combining Phylogenetic and Network Analyses for Orthology Assignment. Mol Biol Evol. 2020;37:3389–96.
3. Simakov O, Marlétaz F, Yue J-X, O’Connell B, Jenkins J, Brandt A, et al. Deeply conserved synteny resolves early events in vertebrate evolution. Nat Ecol Evol. 2020;4:820–30.

**Second round of review**

**Reviewer 1**

This manuscript by Brasó et al. is considerably improved. However, some additional clarification, particularly in the figure captions, is needed for readers who are not hard-core bioinformaticians. One or more of the authors who is not a bioinformatician should have read the manuscript to make sure that it is reasonably clear to the non-specialist. A glossary with definitions of all the specialized terms would help. It could include definitions for words which many, but not all, readers would understand such as “Ohnologs” to those which no one other than a bioinformatician would understand such as “TPM” to those which mean one thing to a bioinformatician and another thing to everyone else such as “intersection.”
A paper usually stands or falls on the quality of its illustrations. Unfortunately, the figures are not clearly labeled and the figure captions do not adequately explain them. The main difficulties are spelled out below.
Figure 2B should be clarified for the non-mathematician. The reader should not have to guess what it shows. For example, I would guess that the 3rd bar shows below the horizontal line the percentages of single-copy genes in B. lanceolatum that are expected to correspond to single copy, Ohnologs and small-scale duplicates in four vertebrates. Above the horizontal line, the 3rd bar apparently shows the single-copy, Ohnologs and small-scale duplications in the four vertebrates expected to correspond to the small-scale duplicates in B. lanceolatum. Presumably the expected values derive from a calculation based upon the single copy genes being randomly distributed among ohnologs, single-copy and small-scale duplicated genes in vertebrates. The fourth bar evidently shows the real percentages. However, this is just a guess at what I think the Fig. 2B indicates. I have no idea what is meant by “intersection between B. lanceolatum and vertebrates.” Do the authors mean “correspondence?” According to Wikipedia “In mathematics, the intersection of two sets A and B is the set containing all elements of A that also belong to B.” So the term “intersection” makes perfect sense to a mathematician, but not to others, who think of intersections as two lines meeting. Thus, to a non-mathematician “the intersection between B. lanceolatum and vertebrates” might well signify the node in the phylogenetic tree where the two lineages split, which makes no sense at all in the context of the figure. I suggest either changing “intersection” to “correspondence” in the figure or including an explanation of “intersection” in the figure caption or glossary for the non-specialist. I don’t know what the arrows are intended to show. There is no explanation of them in the caption. Why is the ratio between observed and expected shown only for the small-scale duplicated and ohnologs corresponding to the small-scale duplicated genes in B. lanceolatum and only for the single copy genes corresponding to the single-copy genes in B. lanceolatum?

For Figure 3, there is no explanation for some dots being light grey and others black. This difference does not seem to be related to the size of the dots.

For Figure 4, what is meant by “Mean gene expression” and why is it termed “TPM” in the caption. I looked up the term. It means “transcripts per million.” So, does 100 on the graph in A and B correspond to 100 million total transcripts? But I learn that the length of a gene is taken into consideration in calculating TPM—presumably because longer genes would be expected to result in more reads for a given level of expression. Presumably the figure takes into consideration that different amounts of RNA may have been sequenced for different tissues and for corresponding tissues from different organisms. This information is not spelled out in the Materials and Methods section or in the figure caption and would be useful to have for those not familiar with the techniques. I assume that the bars in Figure 4 represent the full extent of expression of all the single copy and duplicate genes and that the vertical lines (whiskers?) are something akin to the standard error of the mean or standard deviation. It would be useful to include the information in the figure caption. In equating the tissues in amphioxus and vertebrates, there is one problem, probably not vital for the present study, that the so-called “liver” in amphioxus, which is simply a diverticulum of the gut that is evidently capable of absorbing food, is probably not homologous to the “liver” in vertebrates.

Figure 5 refers to “conditions with expression.” The top figure hints that “condition” might mean “tissue.” However, the definition of “condition” is “the state of something with regard to its appearance, quality, or working order” or “the circumstances affecting the way in which people live or work, especially with regard to their safety or well-being.” Conditions simply aren’t tissues. If “tissue” is what the authors mean, why don’t they say “tissue.” I can only guess that in some mathematical formula one variable is termed a “condition.” Secondly, the Y- axes in the lower part of the figure aren’t labeled. By the “union of both duplicates expression” it looks from the figures on the right that they mean all the tissues in which either of the duplicates is expressed. If that is the case, the authors should say so.
Figure 6. Although the number of chromosomes in the two species is stated in the text, the authors should indicate on the axes of the figure that the numbers 1-19 refer to chromosomes. The figure caption should state the significance of the different colors. I think that the different colors are used to indicate the genes on each of the chromosomes since the lines separating one chromosome from another are faint. If this is true, the authors need to say so in the caption.
In sum, while the authors have redone the figures, they apparently did not subject them to the scrutiny of a reader who is not steeped in bioinformatics. I do hope my comments above will show them how they can reach that reader.
In regard to the text, it is much more readable. I am not sure how the Broccoli algorithm is able to distinguish duplicates occurring before the split of amphioxus and vertebrates, from those occurring independently at the base of each of the two lineages, but I will take the authors’ word that it can make that distinction.
The English is generally acceptable, but I will suggest a few improvements. In general, it is best to avoid long strings of adjectives modifying a noun. Thus, on p. 3, line 16/17 I suggest changing “chordate ancestor genome structure” to “the ancestral genome structure of chordates.”
p. 3 line 28. “their” would have to refer to “duplications” which is the nearest plural noun. That is nonsense. “their” should be changed to “its” to refer to “the vertebrate lineage” which is singular, not plural.
p. 3 line 31. Change “contribution” to “contributions”.
p. 3. Line 48. Reference 1, only sampled Branchiostoma lanceolatum populations in the northeastern Atlantic and the Mediterranean. The species is also reported from the waters off Iran, East Africa and India. Therefore, the sentence should read “an ecological range extending at least from the northeastern Atlantic…..” The correct word is “extending”, not “expanding.”
p. 3. Line 58. Same problem as on lines 16/17. Should read “the early evolution of chordates.”
p. 3. Line 60/61. Same problem. “gene duplication evolutionary histories” should read the history of gene duplications during evolution of these two lineages.”
p. 4. Lines 27/28. Should read “the quality of BraLan3 is comparable to that of vertebrate genome assemblies….” Please eliminate the terms “model species” and “non-model” species. They are meaningless terms designed to inflate or, in the case of non-model, to deflate the importance of the species studied.
p. 4 lines 54/55. Fig. 1 caption. There is not a single species of amphioxus off the American or Asian coasts. Therefore, for B. floridae, the authors could say “an American amphioxus” or “the Florida amphioxus.” For B. belcheri, they should say “an Asian amphioxus.”
p. 5, llines 56/57. Same problem as in Fig. 1 caption. Also change “vertebrate model species” to “vertebrates”—saves space.
p. 8. It might be useful for the general reader if “orthogroup” were defined. One and perhaps the only definition is “set of genes derived from a single gene in the last common ancestor.”
p. 9. Line 38. Italicize B. lanceolatum.
p. 17, line 26. Please eliminate “non-model.”
p. 18. Line 36/37. “scaffold” is not a verb. Best to say “In addition, we divided the resulting haploid assembly into scaffolds….”
p. 24. As of this review, reference 11, Huang et al, has not yet been published. As bioRxiv is not a peer-reviewed publication, manuscripts posted there should not be cited until they are published in a peer-reviewed journal.
Reference list in general. Please be consistent in capitalizing words in titles. In some instances, the authors have capitalized all the words except the articles and in others they haven’t. They should follow the style for the journal. There is no excuse for such inconsistency.

**Reviewer 2**

I’d like to thank the authors for the nice effort at answering my comments. The manuscript is much improved, especially with regards to the figures.

I have a couple of minor suggestions that can be validated at the discretion of the editor.

1) The introduction would benefit from proof-reading and tightening. For example the sentence “Unlike vertebrate genomes, only small-scale gene duplicates are present in the amphioxus genome, but their number and functional contribution are unknown.” Is redundant with the previous one and makes for fastidious reading.
2) Figure 2B: the meaning of the horizontal black line across the 3rd and 4th columns is not given, yet seems crucial to understand which we see each shade of green twice.

**Authors’ response to reviewers**

Reviewer #1:
This manuscript by Brasó et al. is considerably improved. However, some additional clarification, particularly in the figure captions, is needed for readers who are not hard-core bioinformaticians. One or more of the authors who is not a bioinformatician should have read the manuscript to make sure that it is reasonably clear to the non-specialist. A glossary with definitions of all the specialized terms would help. It could include definitions for words which many, but not all, readers would understand such as "Ohnologs" to those which no one other than a bioinformatician would understand such as "TPM" to those which mean one thing to a bioinformatician and another thing to everyone else such as "intersection."
Response:
While we understand the reviewer's concerns, we have explained the necessary terms when they are used and added additional explanations in this revision. This terminology is standard for a genome journal such as Genome Biology, and thus we do not feel that adding a glossary in addition to the definitions already provided within the text would be appropriate. Furthermore, it is not clear to us where a Glossary section would fit within the journal formatting style. In any case, we defer to the Editor's opinion, and we would be happy to follow his recommendations on this question.

A paper usually stands or falls on the quality of its illustrations. Unfortunately, the figures are not clearly labeled and the figure captions do not adequately explain them. The main difficulties are spelled out below.

Figure 2B should be clarified for the non-mathematician. The reader should not have to guess what it shows. For example, I would guess that the 3rd bar shows below the horizontal line the percentages of single-copy genes in B. lanceolatum that are expected to correspond to single copy, Ohnologs and small-scale duplicates in four vertebrates. Above the horizontal line, the 3rd bar apparently shows the single-copy, Ohnologs and small-scale duplications in the four vertebrates expected to correspond to the small-scale duplicates in B. lanceolatum. Presumably the expected values derive from a calculation based upon the single copy genes being randomly distributed among ohnologs, single-copy and small-scale duplicated genes in vertebrates. The fourth bar evidently shows the real percentages. However, this is just a guess at what I think the Fig. 2B indicates. I have no idea what is meant by "intersection between B. lanceolatum and vertebrates." Do the authors mean "correspondence?" According to Wikipedia "In mathematics, the intersection of two sets A and B is the set containing all elements of A that also belong to B." So the term "intersection" makes perfect sense to a mathematician, but not to others, who think of intersections as two lines meeting. Thus, to a non-mathematician "the intersection between B. lanceolatum and vertebrates" might well signify the node in the phylogenetic tree where the two lineages split, which makes no sense at all in the context of the figure. I suggest either changing "intersection" to "correspondence" in the figure or including an explanation of "intersection" in the figure caption or glossary for the non-specialist. I don't know what the arrows are intended to show. There is no explanation of them in the caption. Why is the ratio between observed and expected shown only for the small-scale duplicated and ohnologs corresponding to the small-scale duplicated genes in B. lanceolatum and only for the single copy genes corresponding to the single-copy genes in B. lanceolatum?

Response:
After the first revisions of this manuscript and aiming to resolve the concerns of this reviewer we carefully studied how to make all the figures but specially the present Figure 2B more clear and understandable by the general public. We are sure that the reviewer can appreciate how much it has improved since its first version. Nevertheless, we agree with the reviewer that the figure and its caption still had room for improvement. We redesigned Figure 2A to allow for a more clear connection between 2A and 2B, and revised and extended the explanation on how to interpret columns 3 and 4 of Figure 2B. We also clarified which categories are depicted with arrows between these two columns.
With respect to the term “intersection”, we understand that some of the audience might not be stepped into bioinformatics and that is very important to adapt the text and figures to their level of expertise. Nevertheless, we find that the use of the term “intersection” in the mathematics set theory is very widespread, commonly used and known. The evidence of this is that a quick search on Wikipedia has given this reviewer the clue to understand this specific usage of the word “intersection”. We also think that precision is a very important characteristic of scientific writing, and that the term “intersection” depicts with precision what is shown in columns 3 and 4 of Figure 2B. For all these reasons, we have decided to continue using the word “intersection” in such context and to clarify that “intersection” refers to “the intersection of sets” this specific context in the figure caption.
We think that this figure has greatly improved in clarity and is much more accessible to a broad public thanks to this reviewer's comments since the beginning. We would like to thank the reviewer for this, and we hope these modifications satisfy the reviewer’s concerns about this figure.

For Figure 3, there is no explanation for some dots being light grey and others black. This difference does not seem to be related to the size of the dots.
Response:
All points in figure 3 are colored the same semi-transparent black. The apparent differences in color are due to the superposition of dots in some areas. That is, point density can be appreciated, to a certain extent, through color darkness. We have added a clarification in the caption. Thank you for noticing it was not clear.

For Figure 4, what is meant by "Mean gene expression" and why is it termed "TPM" in the caption. I looked up the term. It means "transcripts per million." So, does 100 on the graph in A and B correspond to 100 million total transcripts? But I learn that the length of a gene is taken into consideration in calculating TPM—presumably because longer genes would be expected to result in more reads for a given level of expression. Presumably the figure takes into consideration that different amounts of RNA may have been sequenced for different tissues and for corresponding tissues from different organisms. This information is not spelled out in the Materials and Methods section or in the figure caption and would be useful to have for those not familiar with the techniques. I assume that the bars in Figure 4 represent the full extent of expression of all the single copy and duplicate genes and that the vertical lines (whiskers?) are something akin to the standard error of the mean or standard deviation. It would be useful to include the information in the figure caption. In equating the tissues in amphioxus and vertebrates, there is one problem, probably not vital for the present study, that the so-called "liver" in amphioxus, which is simply a diverticulum of the gut that is evidently capable of absorbing food, is probably not homologous to the "liver" in vertebrates.
Response:
Transcripts per million or TPM is a commonly used measure of gene expression that corrects for the length of the transcripts and is designed to be comparable between samples. A gene with a value of 100 TPM on any of Figure 4 plots means that for every 1 million reads in a sample, 100 corresponded to this specific gene. So, yes, the used measure corrects by transcript different lengths and different amounts of RNA molecules in different samples. We added a clarification of the acronym “TPM” in the figure caption and an explanation of this gene expression measure in Methods.
The plots in Figure 4 are box plots. They are commonly used in many areas of biological research. We have added a clarification in Figures’ 4 and S3 captions.
We are aware that we are matching amphioxus organs with their zebrafish most close homologous organ and that some matches might not be extremely straightforward but, as the reviewer says, we don’t think that these differences affect our results.

Figure 5 refers to "conditions with expression." The top figure hints that "condition" might mean "tissue." However, the definition of "condition" is "the state of something with regard to its appearance, quality, or working order" or "the circumstances affecting the way in which people live or work, especially with regard to their safety or well-being." Conditions simply aren't tissues. If "tissue" is what the authors mean, why don't they say "tissue." I can only guess that in some mathematical formula one variable is termed a "condition." Secondly, the Y- axes in the lower part of the figure aren't labeled. By the "union of both duplicates expression" it looks from the figures on the right that they mean all the tissues in which either of the duplicates is expressed. If that is the case, the authors should say so.
Response:
We have discussed the word to use in Figure 5 to refer to the considered entities, with both experimental and bioinformatician biologists. We couldn’t use the term “tissue” because not all cases are tissues, one of the entities is actually a developmental stage (embryonic whole-body). We chose the word “condition” as in “experimental condition” or in other words, the procedure/treatment/variable that changes between different samples or measurements of an experiment/analysis. Given the context, we think that the choice of the term “condition” is adequate. This is why we decided to keep this term, adding a clarification in the figure caption.
We labeled the Y-axes.
We clarified the definition of union of both duplicates' expression in Figure 5 caption as the reviewer proposed. It is more clear now, thank you for proposing it.

Figure 6. Although the number of chromosomes in the two species is stated in the text, the authors should indicate on the axes of the figure that the numbers 1-19 refer to chromosomes. The figure caption should state the significance of the different colors. I think that the different colors are used to indicate the genes on each of the chromosomes since the lines separating one chromosome from another are faint. If this is true, the authors need to say so in the caption.
Response:
We have clarified the labels of all gene synteny figures and have added an explanation for the point colors in the figure captions.

In sum, while the authors have redone the figures, they apparently did not subject them to the scrutiny of a reader who is not steeped in bioinformatics. I do hope my comments above will show them how they can reach that reader.
In regard to the text, it is much more readable. I am not sure how the Broccoli algorithm is able to distinguish duplicates occurring before the split of amphioxus and vertebrates, from those occurring independently at the base of each of the two lineages, but I will take the authors' word that it can make that distinction.
Response:
Thank you for appreciating the effort we had done in making the text clearer and more accessible to a broader audience.
Regarding Broccoli, it is a well recognized algorithm published 2 years ago with already 28 citations that use it to find orthologous groups. Broccoli was specially designed to split orthogroups with duplications more ancient than the last common ancestor of all the considered species. In the previous round of revisions and following a concern of another reviewer, we implemented an independent method validating 80% of the concerned cases (small-scale duplicates in both branches). The validation results were added to the manuscript. We consider this concern to have objectively been addressed.

The English is generally acceptable, but I will suggest a few improvements. In general, it is best to avoid long strings of adjectives modifying a noun. Thus, on p. 3, line 16/17 I suggest changing "chordate ancestor genome structure" to "the ancestral genome structure of chordates."
Response:
Done.
p. 3 line 28. "their" would have to refer to "duplications" which is the nearest plural noun. That is nonsense. "their" should be changed to "its" to refer to "the vertebrate lineage" which is singular, not plural.
Response:
Changed. Thank you for noticing the error.
p. 3 line 31. Change "contribution" to "contributions".
Response:
Rephrased sentence.
p. 3. Line 48. Reference 1, only sampled Branchiostoma lanceolatum populations in the northeastern Atlantic and the Mediterranean. The species is also reported from the waters off Iran, East Africa and India. Therefore, the sentence should read "an ecological range extending at least from the northeastern Atlantic….." The correct word is "extending", not "expanding."
Response:
Done.
p. 3. Line 58. Same problem as on lines 16/17. Should read "the early evolution of chordates."
Response:
Done.
p. 3. Line 60/61. Same problem. "gene duplication evolutionary histories" should read the history of gene duplications during evolution of these two lineages."
Response:
Changed.
p. 4. Lines 27/28. Should read "the quality of BraLan3 is comparable to that of vertebrate genome assemblies…." Please eliminate the terms "model species" and "non-model" species. They are meaningless terms designed to inflate or, in the case of non-model, to deflate the importance of the species studied.
Response:
Changed.
p. 4 lines 54/55. Fig. 1 caption. There is not a single species of amphioxus off the American or Asian coasts. Therefore, for B. floridae, the authors could say "an American amphioxus" or "the Florida amphioxus." For B. belcheri, they should say "an Asian amphioxus."
Response:
Adapted, thank you.
p. 5, llines 56/57. Same problem as in Fig. 1 caption. Also change "vertebrate model species" to "vertebrates"—saves space.
Response:
Done.
p. 8. It might be useful for the general reader if "orthogroup" were defined. One and perhaps the only definition is "set of genes derived from a single gene in the last common ancestor."
Response:
Included. Thanks for the suggestion.
p. 9. Line 38. Italicize B. lanceolatum.
Response:
Done.
p. 17, line 26. Please eliminate "non-model."
p. 18. Line 36/37. "scaffold" is not a verb. Best to say "In addition, we divided the resulting haploid assembly into scaffolds…."
Response:
To scaffold can be a verb https://www.dictionary.com/browse/scaffold .
p. 24. As of this review, reference 11, Huang et al, has not yet been published. As bioRxiv is not a peer-reviewed publication, manuscripts posted there should not be cited until they are published in a peer-reviewed journal.
Response:
Genome Biology policy, like that of most journals, accepts citation of preprints:
“Only articles, clinical trial registration records and abstracts that have been published or are in press, or are available through public e-print/preprint servers, may be cited.”
From https://genomebiology.biomedcentral.com/submission-guidelines/preparing-your-manuscript
Reference list in general. Please be consistent in capitalizing words in titles. In some instances, the authors have capitalized all the words except the articles and in others they haven't. They should follow the style for the journal. There is no excuse for such inconsistency.
Response:
We have revised with caution the list of references and its format.


Reviewer #2:
I'd like to thank the authors for the nice effort at answering my comments. The manuscript is much improved, especially with regards to the figures.

I have a couple of minor suggestions that can be validated at the discretion of the editor.

1) The introduction would benefit from proof-reading and tightening. For example the sentence "Unlike vertebrate genomes, only small-scale gene duplicates are present in the amphioxus genome, but their number and functional contribution are unknown." Is redundant with the previous one and makes for fastidious reading.
Response:
We have given attention to the introduction text, including removing the concerning sentence. It has substantially improved. Thank you for suggesting it.
2) Figure 2B: the meaning of the horizontal black line across the 3rd and 4th columns is not given, yet seems crucial to understand which we see each shade of green twice.
Response:
We clarified the meaning of the horizontal line in Figure 2B and revised the whole figure to make it more understandable.
